# Understanding of Leaf Development—the Science of Complexity

**DOI:** 10.3390/plants2030396

**Published:** 2013-06-25

**Authors:** Robert Malinowski

**Affiliations:** Polish Academy of Sciences Botanical Garden-Centre for Biodiversity Protection in Powsin, ul Prawdziwka 2, 02-973 Warsaw, Poland; E-Mail: r.malinowski@obpan.pl; Tel.: +48-22-754-2610

**Keywords:** leaf morphogenesis, biophysical and cellular aspects of leaf development, leaf shape

## Abstract

The leaf is the major organ involved in light perception and conversion of solar energy into organic carbon. In order to adapt to different natural habitats, plants have developed a variety of leaf forms, ranging from simple to compound, with various forms of dissection. Due to the enormous cellular complexity of leaves, understanding the mechanisms regulating development of these organs is difficult. In recent years there has been a dramatic increase in the use of technically advanced imaging techniques and computational modeling in studies of leaf development. Additionally, molecular tools for manipulation of morphogenesis were successfully used for *in planta* verification of developmental models. Results of these interdisciplinary studies show that global growth patterns influencing final leaf form are generated by cooperative action of genetic, biochemical, and biomechanical inputs. This review summarizes recent progress in integrative studies on leaf development and illustrates how intrinsic features of leaves (including their cellular complexity) influence the choice of experimental approach.

## 1. Introduction

The emergence of leaves has had a tremendous impact on the entire planet [1]. During the course of evolution, leaves have developed certain forms and shapes in order to adjust to the environment or to maximize life strategies and propagation. Today, understanding the mechanisms regulating leaf development has two main points; the first is related to gathering the biological knowledge and the second is related to the future potential of engineering energy efficient plants with increased biomass production. In order to achieve this, we must characterize all of the factors involved in the development of leaf shape and size and gain an understanding of how they are interconnected. This is a difficult task, mainly due to the fact that leaves are multicellular organs and their morphogenesis and growth are regulated in a complex manner. Frequently, understanding leaf biology has been limited by the narrow focus of individual studies. Due to the very dynamic progress of molecular approaches, the accepted scientific thinking has often resulted in publications focusing on individual genes and proteins rather than focusing on an integrated view of the whole process. Despite the obvious scientific value of such work, the derived conclusions were frequently misleading or not sufficient to fully illustrate the complex biological issues related to leaf biology. Taking into account the growing evidence on complex coordination between biophysical and molecular regulatory mechanisms during plant organogenesis, a return to the old research paradigm where observation of the phenomenon drives further direction of detailed studies may strengthen our understanding of leaf development. Once combined with modern techniques of cell visualization, mathematical modeling, and image analysis this approach may be very helpful for deciphering global rules governing leaf shape and size development. This research approach has recently become more popular, resulting in several interesting outcomes. Therefore, we decided to summarize and illustrate how interdisciplinary methods have contributed to our understanding of leaf development. Another useful approach for integrative studies on leaf biology is the targeted manipulation of morphogenesis. The basic principle of this method is the use of all available methods to modify processes such as cell growth, expansion, or division. This modification is usually performed with the help of chemical factors or changes in the concentration or location of proteins that specifically influence cell division or growth. Manipulation of morphogenesis may also include the application of compressive force or cell ablation techniques. In this review, we are aiming to summarize how integrative studies based on defined local modification of cellular parameters, combined with accurate cell imaging techniques, can facilitate a greater understanding of leaf morphogenesis. This approach has remained unexplored for many years and only recently has technical progress enabled us to obtain a more holistic view of leaf development.

Since this review will frequently refer to biophysical issues we would like to introduce two terms, which will be used to describe specific phenomena. “Stress” describes the force acting upon tissue and “Strain” is the degree of deformation caused by “stress”. Using these terms helps to describe how multicellular organisms are subjected to physical forces and how they maintain cellular integrity. For an in-depth review of plant biomechanics, refer to Boudaud [2]. 

Changes in the mechanical properties of particular cells and tissues occur throughout the life of a plant and are necessary for adaptation to the environment. Since all cells in plants are connected, they work together and each single change of biophysical properties may significantly affect the fate of the entire organ. Leaves are primarily composed of an external layer of epidermal cells, several layers of different types of parenchyma cells, and conductive tissue. From the biophysical perspective, different cell layers or tissues within the leaf are likely to have different mechanical properties. The three main tissues (epidermis, parenchyma, and conductive tissue) have different growth dynamics and rigidity, as well as the size and shape of cells they are built of. These differences result in the generation of tension, therefore the organ is under constant stress. In fact, some intrinsic features of particular components of the lamina may help to withstand these tensions. For example, a quick look at the epidermal pavement cells show that they are very similar to the pattern of interlocking bricks so successfully used for pedestrian walk-ways. This copy of the Mother Nature idea is not incidental but is related to the fact that interlocking increases the resistance to tension and helps to maintain the structural integrity of the entire layer. Looking at it from this perspective may help to illustrate how leaves grow and maintain their flat structure. On the other hand, understanding how each particular element contributes to this so-called “leaf engineering project” may help future work on the modification of plant architecture.

Since the main focus of this review is to summarize current knowledge on cellular aspects of leaf development and explain how integrative methods helped to resolve complex biological problems related to leaf morphogenesis and subsequent growth, we did not include detailed descriptions of genetic regulation of leaf shape and size development. This subject has been already extensively reviewed. For further reading, follow the references given at the end of this paper [3,4].

## 2. Leaf Primordium Formation—The Biomechanical Perspective

**Figure 1:**
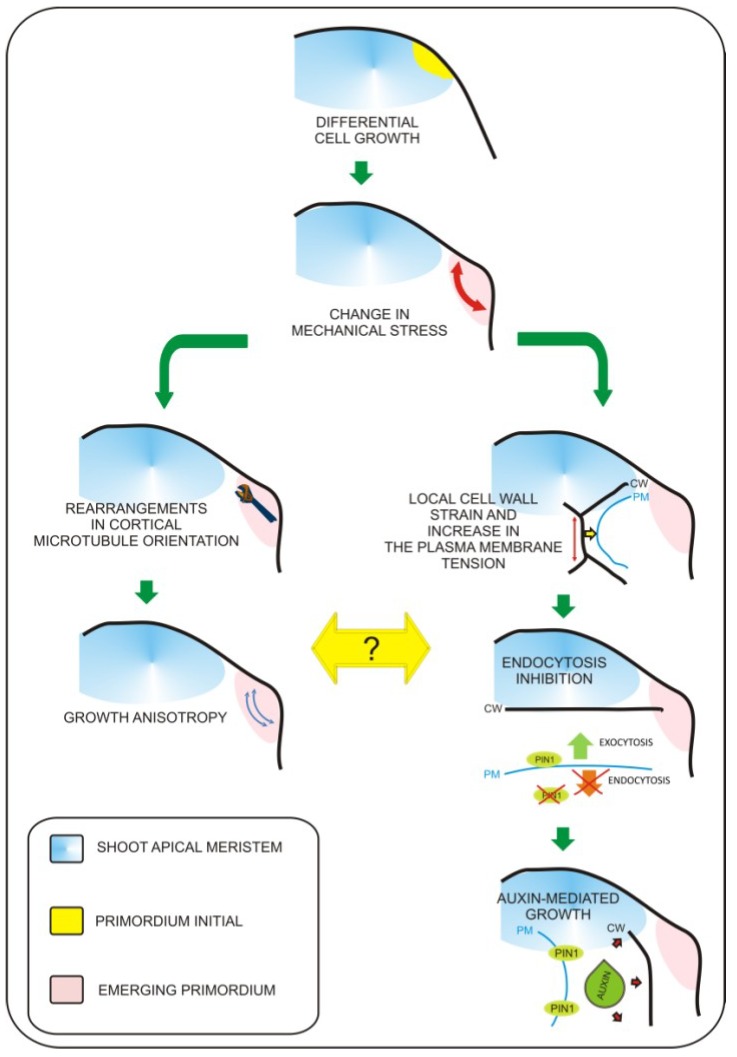
Mechanisms coordinating biomechanical signals with growth responses. Leaf primordium initiation involves local biomechanical changes. So far, two major pathways involved in this process were characterized. The first pathway involves microtubule rearrangements leading to growth anisotropy (left branch). The second is based on the impact of mechanical inputs on plasma-membrane protein trafficking (right branch). Mechanical stress leads to change in PIN1 auxin transporter distribution, this way increasing transport of auxin towards the apoplast. This leads to extracellular space acidification and cell wall loosening, which is required for turgor based growth of cells. So far everything shows that these two pathways are independent. Figure based on recent works of Uyttewaal *et al*. 2012 [14] and Nakayama *et al*., 2012 [15].

Leaf primordium specification occurs at the flank of the shoot apical meristem and depends on the proper gradient of auxin distribution to specify “leaf primordia initials” [5]. Recently, molecular biology studies have revealed pathways that regulate the switch from non-determined to determined growth characteristics for leaf primordium formation, as well as pathways regulating the establishment of basic leaf polarity axes [6]. At present, the exact sequence of events leading to the formation of the future site of leaf initiation is not fully understood. However, the role of biomechanical factors in this process becomes evident. Biophysical factors have long been considered as potential inputs influencing organogenesis within the shoot apex. This was experimentally verified when the cell wall loosening protein expansin was applied to the shoot apex [7]. Expansin application resulted in local changes in cell wall properties that ultimately influenced leaf phyllotaxy and led to differentiation of primordia-like structures. This result clearly demonstrated that local biophysical properties of cells have dramatic impacts on global aspects of organogenesis within the shoot apex. This has been additionally proven by local induction of expansin gene expression in the tetracycline inducible system [8] and recently by overexpression of another cell wall modifying enzyme—pectin methylesterase (PME) [9]. Further progress has been achieved with the development of precise tools for cell imaging and techniques for artificial modification of stress within the shoot apex [10,11]. This work has led to the observation that mechanical forces are involved in microtubule orientation and auxin transport [10,11]. The authors found that microtubule orientation follows stress orientation. This particular pattern of microtubule orientation influences cellulose deposition within the cell wall and is responsible for anisotropic cell growth. The most significant finding in this work is that membrane specific localization of the major auxin transporter PIN1 also depends on stress distribution. Recently, Kierzkowski *et al*. [12] showed that different growth dynamics observed between the central region of the shoot apex and its boundaries (including developing leaf primordia) overlaps with differences in the elastic properties of cells. By application of hyper- or hypo-osmotic solution to particular areas of the apex, Kierzkowski *et al*. demonstrated that the elastic properties of cells influence their expansion or shrinkage upon modification of turgor pressure. The authors suggest that strain-stiffening in the central meristem could be one of mechanisms influencing the balance between meristem self-maintenance and organogenesis. This global mechanical feedback may be additionally influenced by local changes in cell elasticity triggered by cell wall remodeling enzymes. This concept is further supported with experimental evidence from atomic force microscopy (AFM) measurements performed on the shoot apices of the plants with increased amounts of pectin methylesterases (PME) [13]. These observations lead to enormous progress in understanding of the plant morphogenesis. However, due to the fact that the factors responsible for mediating between mechanical inputs and physiological growth responses were unknown, we were still far from understanding how changes in mechanical properties of cells are transferred into physiological responses. In 2012, Uyttewaal *et al*. [14] performed a series of experiments based on local application of a compressive force or cell ablation within different regions of the shoot apex of wild type and the *atktn1 Arabidopsis* mutant. This work has led to the discovery that the microtubule-severing protein called katanin (derived from the word for the Japanese sword, “katana”) regulates cell competence to respond to mechanical stress and may be involved in mediation between mechanical inputs and further growth responses. In addition, Nakayama *et al*. [15] showed how exact biomechanical factors can regulate patterns of auxin transport and distribution within the shoot apex (this phenomenon was previously described by Hamant *et al*. [10]). The main player in this process is the plasma membrane, which can respond to mechanical stimuli by changes in membrane protein turnover. In order to demonstrate this concept, the authors [15] applied local force, triggered changes in cellular turgor pressure, or induced cell growth by apoplast acidification (local auxin or acid treatment). This approach has led to the observation that cells located within the area of deformation contain higher amounts of the PIN1 auxin efflux protein and that local strain positively influences plasma membrane localization of PIN1. From previous experiments, we know that blocking auxin transport by the NPA does not affect cytoskeleton rearrangements [10], therefore, we can speculate that the mechanism by which katanin mediates between mechanical inputs and growth responses is auxin-independent. Taken together, we can say that it becomes clear that precise changes in mechanical properties of cells are key factors influencing leaf morphogenesis within the shoot apex. So far, it seems that mechanical inputs are transferred by at least two parallel pathways (Figure 1). First, morphogen-based regulation is mediated by the plasma membrane and based on changes in auxin transport/distribution leading to differential growth responses. The second mechanism is related to growth anisotropy and is mediated by proteins involved in microtubule rearrangements such as katanin. Further research will be necessary to determine a precise explanation of how these two mechanisms are interconnected.

## 3. Light Directed Leaf Morphogenesis—The Cellular Response

In recent years, much effort has been devoted to the study of the impact of light on leaf morphogenesis. The presence of light is necessary for leaf primordia formation; however, no exact mechanism for this regulation has been provided. One very important event accompanying early steps of leaf differentiation is the acquisition of photosynthetic capability by the developing leaf primordium. Transcriptional profiling has shown that expression of genes related to carbohydrate metabolism accompanies the early steps of leaf differentiation [16]. It is quite intuitive to consider that light must be involved in leaf differentiation, particularly because the lack of light negatively influences leaf primordia formation. Yoshida *et al*. [17] showed however, that light-dependent primordium formation is not regulated by any long distance signaling pathways related to photosynthesis but by the local regulation and balance between auxin and cytokinin within the shoot meristem. There is a high probability that at least some part of this mechanism is related to the spatio-temporal regulation of cell proliferation. It follows then, that understanding the exact relation between auxin and cytokinin, and its impact on cellular events within the shoot apex may help to integrate current knowledge. At present, we know that formation of new primordium requires oriented cell divisions [18], but it remains unclear how precise regulation of cell cycle progression within different areas of shoot apex contributes to leaf organogenesis. So far, experiments based on modification of cell cycle-related genes led to contradictory results [19,20,21], therefore, future work on this subject may require the use of precise regulatory elements to restrict and define cell cycle modification to a particular cellular context. 

## 4. Epidermis and Mechanical Regulation of Turgor-Driven Leaf Expansion

Epidermal tissue can work as a growth rate limiting factor influencing turgor pressure-based leaf lamina expansion, as shown in experiments in which expression of a brassinosteroid receptor gene *BRI1* in the epidermis of a *bri1* mutant restored normal leaf size [22]. The BRI1 receptor is the major protein involved in perception of the brassinosteroids at the plasma membrane. Phosphorylation of its intracellular domain triggers transduction of the brassinosteroid signal and leads to physiological responses, including cell growth [23]. The *bri1* mutation leads to extreme dwarfism caused by significant reduction of cellular growth [24]. Savaldi-Goldstein *et al*. [22] showed that the expression of *BRI1* in the epidermis of the *bri1* genetic background was sufficient to restore proper leaf growth. What is even more important is that this resulted in normal parenchyma cell growth. Despite the fact that this experiment shows that epidermal growth is a major factor responsible for lamina expansion, it remains unknown whether this observed phenomenon is related only to mechanical changes in the epidermis or other BR signaling related responses. At the organ level, epidermal growth has been modified by overexpression of the KRP1/ICK1 protein [25]. The KRP1 protein is a negative factor involved in the G1-S cell cycle progression [26]. Depending on the protein concentration, it may block G1-S transition, resulting in cell division arrest, or blocking entry into mitosis with simultaneous S phase progression leading to endoreduplication [27]. An increased level of this protein causes decreases in cell number and lamina size [28,29]. One characteristic effect of KRP1 increase is the development of abnormally large cells, which may be related to the fact that cells are connected and a certain degree of compensation occurs. This hypothesis coincides with the fact that increased levels of KRP1 in the trichome cell (which grows independently from internal lamina cells) reduced its size [30]. Overexpression of *KRP1* in the epidermis decreased lamina size, demonstrating that growth in the L1 layer influences the entire organ [25]. Conversely to results obtained by Savaldi-Goldstein *et al*., [22] the epidermal expression of the *KRP1* gene did not influence the internal cell layer, since no increase in cell size within the internal layer was observed. To gain a better understanding of this phenomenon, future experiments may require modification of leaf growth by epidermal overexpression of factors facilitating cell wall extensibility or remodeling. This would help to distinguish between clearly biophysical and physiological effects of epidermal growth. We believe that further understanding of the cellular dynamics within the epidermis will benefit scientists working on various aspects of plant developmental biology and leaf form engineering.

## 5. Proliferation/Differentiation Balance within Leaf Epidermis

**Figure 2:**
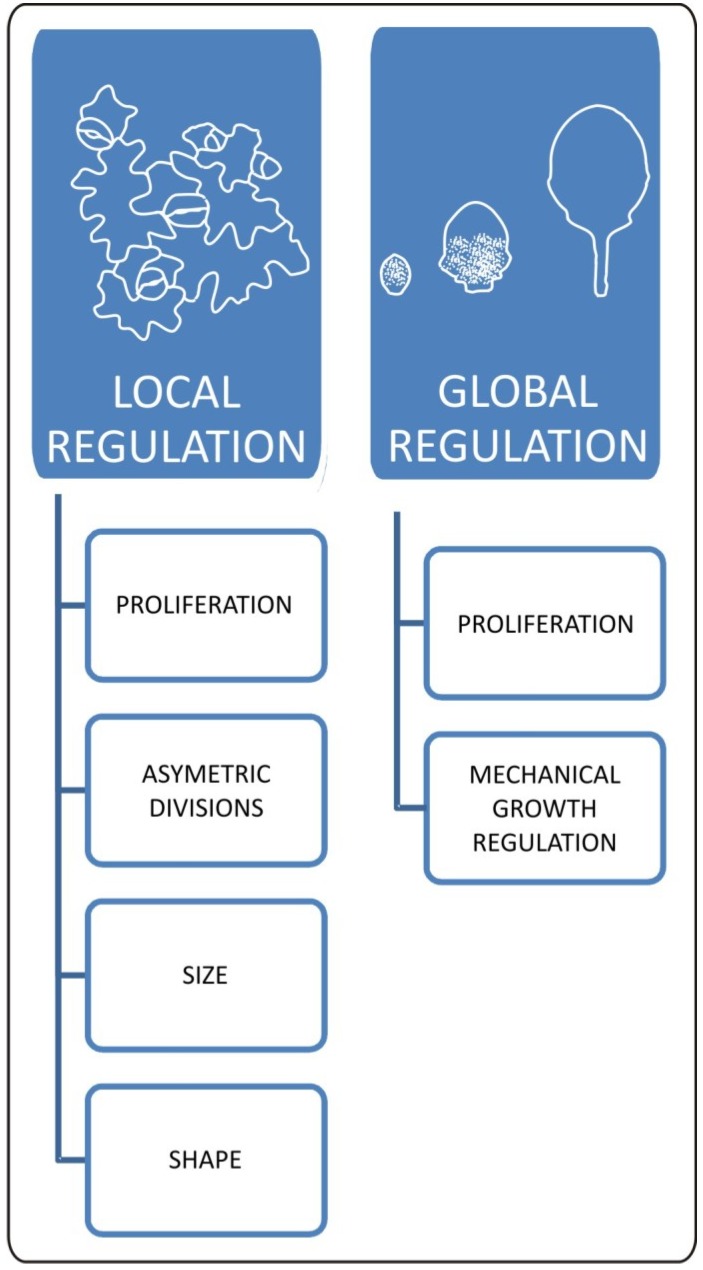
Local and global aspects of changes in epidermal properties during leaf development. Cellular changes within epidermis have a huge impact on final leaf form. Locally mechanisms regulating meristemoid cell fate influence distribution of stomata and pavement cells. This process has a huge impact on overall plant growth responses as well. Size and shape of pavement cells is the outcome of interaction between biomechanical and genetic factors. These local cellular changes are strictly connected with global effect of epidermis on leaf development. At the organ level, epidermis works as a mechanical growth rate-limiting factor. Epidermal growth and final leaf form is influenced by genetic and physiological inputs regulating patterns of cell proliferation.

As we mentioned above, the epidermis has a large impact on leaf growth and final form acquisition, therefore it is very important to understand the mechanisms regulating cellular dynamics within this tissue. Within the epidermis, we can distinguish cells of strikingly different form: puzzle piece-shaped pavement cells, guard cells, meristemoid cells, trichomes, and long margin cells. Differentiation of these cells is regulated via specialized pathways that influence microtubule reorganization, endoreduplication and differential cell wall synthesis or modification. The majority of cells that make up the leaf surface are generated via asymmetric divisions of meristematically active cells called meristemoids [31]. Due to the fact that certain factors determine the polarity of meristemoid mother cell divisions, and their further cell fate in a transient fashion, precise and non-invasive techniques must be applied in order to study cell proliferation/differentiation balance within the epidermis. Recently, cell tracking and time-lapse imaging techniques were used to determine how the timing of cell cycle progression and the precise regulation of the polarity of cell divisions are crucial for epidermal development [32,33]. These studies demonstrated that the maintenance of epidermal meristematic activity depends on the ability to retain the speechless (SPCH) transcription factor, whereas polar localization of the BASL [32] and POLAR [33] proteins provides positional information for the determination of which daughter cells retain meristematic activity after asymmetric division. In addition, transcriptional profiling showed that increased levels of 17 core cell cycle genes were present in a meristemoid-enriched fraction [33]. Interestingly, the authors also found that elements of cytokinin signaling (*ARR16*), catabolism (*CKX4*), and cytokinin related differentiation regulation (*CLE9*) were also upregulated in the meristemoid-enriched fraction. This suggests the existence of a precise cytokinin-related mechanism regulating the timing of cell proliferation and maintenance of meristemoid cells in an undifferentiated state. The potential involvement of hormonal factors in the maintenance of meristematic activity within the epidermis is additionally supported by the fact that cell divisions are distributed in the longitudinal gradient from tip to base during early steps of leaf primordium development [34]. The existence of such a pattern shows that cell proliferation must be globally coordinated at the organ level (Figure 2) and phytohormones like auxin or cytokinin have all the necessary features to be involved in this process. Recently, this phenomenon has been more extensively studied and more abrupt cell cycle arrests along the proximo-distal axis were reported [35]. What is interesting is that the authors found a positive correlation between achievement of cellular photosynthetic capacity (retrograde signaling coordinating plastid protein import and nuclear gene expression) and transition towards cell enlargement. This shows that other physiological inputs may coordinate cell proliferation during leaf development.

Epidermal cell division, growth, and expansion occur simultaneously during several stages of leaf development. The main challenge that lies before us is to properly separate and describe these processes. At least partially, this can be achieved via a combination of modeling and kinematic observation approaches. Such work has been published recently by Kheibarshekan *et al*. and led to the discovery of many interesting details on cellular events within epidermis [36]. At first, the authors showed that cell cycle duration in leaf epidermis is constant for all types of cells reaching the approximate value of 20 hours [36]. They also found that the maximum growth rate for pavement cells coincides with the early leaf expansion phase at which cell proliferation still occurs. Moreover, kinematic observations showed that small pavement cells grow faster than the large ones and, in general, cells within the leaf epidermis of the same organ grow at different rates. This has been further demonstrated with the help of the sequential replicas and scanning microscopy method by Elsner *et al*. [37]. Kheibarshekan *et al*. [36] pointed out that the highest growth rate of small pavement cells is most probably generated by the endoreduplication process since increases in cellular DNA content can be observed at that moment. In late stages of leaf development, pavement cells grow more slowly. However disproportion in growth rate between smaller and larger pavement cells is maintained. The obvious implication of this uneven growth rate is a generation of mechanical stress. Observations made by Kheibarshekan *et al*. [36] showed also that in wild type plants, cell divisions are largely independent from cell growth and expansion, suggesting that mechanisms regulating cell cycle progression exclusively influence the maintenance of meristematic activities, proliferation/differentiation balance, and the supply of new cells. As we can see, there are multiple mechanisms involved in local and global coordination within epidermis and they are crucial for the final leaf form (Figure 2).

## 6. Integrating Cellular Balance with General Patterns of Leaf Development

The final leaf size results from a combination of cell number, cell size and growth. To a large extent, the size of the leaf blade is determined by genetic information. Recent work has shown that the timing and maintenance of meristematic activities, and proliferation/differentiation balance within leaf blade, is crucial for the final leaf size (review in [3]). This genetic regulation, however, is strictly connected with intrinsic mechanisms influencing cellular responses. The disturbance of one factor usually leads to a response, which at least partially neutralizes the possible impact of such an action at the organ level. This phenomenon is commonly called compensation, and the work of Ferjani *et al*. [38] showed that various cellular events may lead to it. After a very detailed analysis of cellular interplay in the *an3-4* mutant, the *KRP2-OE* (overexpression) line, and mutations in different alleles of the *FUGU* gene, the authors found that cell enlargement in leaves with a smaller number of cells may be related to increases in size during the mitotic phase, postmitotic increases in cell growth rate, or prolonged cell growth. This result shows that many levels of regulation exist and care must be taken when experiments with mutants of single factors displaying changes in leaf morphology are interpreted. The technique, which is particularly helpful for studying these cellular aspects of leaf size determination, is the chemically inducible manipulation of gene expression where the products directly regulate cell cycle progression. Results of such experiments showed that some existing views on the cellular aspects of leaf size determination must be changed. For example, the frequently proposed mechanism in which increases in cell divisions leads to an increase in leaf size turned out to be a gross over-simplification, since artificially increased cell number did not correlate with increase of blade size [29]. In fact, delayed or inhibited termination of proliferation by the overexpression of the positive regulator of G1-S progression *CYCD3.1* and the silencing of the negative regulator of G1-S progression *RBR1* led to an overall decrease in leaf blade size [29]. This result also shows that despite increased cell division it is important for leaf blade enlargement, the quit of the proliferation state is crucial for this process. This maintains agreement with molecular work where factors (SHORT-ROOT, SCARECROW and SCRAMBLED/STRUBBELIG) regulating maintenance of meristematic activities and proliferation/differentiation balance within the leaf blade were characterized [39,40]. 

So far, high resolution experiments describing spatial and temporal growth patterns exclusively within internal cell layers have not been performed, therefore, we do not exactly know how parenchymatic cells are influencing leaf development. Recent molecular work shows that precise mechanisms regulating cellular dynamics within this layer exist, and at least during early stages of leaf primordium development, internal cell layers may strongly influence lateral leaf blade expansion [41]. The authors provide evidence that expression of two *WOX* genes within the middle region regulates lateral outgrowth of leaf blade expansion. Finally, they suggest that *WOX* genes can modulate activity of the *KLUH* gene encoding cytochrome 450 monooxygenase. *KLUH* has been found to prevent the arrest of cell proliferation in a non-cell autonomous manner, and its mutation leads to decrease in leaf lamina size [42]. Work of Nakata *et al*. [41] suggests that *WOX* genes expression in a middle domain may be a part of important mechanisms, which allow coordination of growth between internal and external cell layers during early stages of leaf development. These experiments show that parenchyma cells may play roles beyond their structural role, therefore modification of cell growth or cell divisions exclusively within internal cell layers combined with the recently developed technique for 3-D observation of subepidermal layers [43,44] needs to be performed for future understanding of cellular interplay during different stages of leaf development. 

## 7. Effects of Vasculature on Leaf Expansion and Final Form Acquisition

Understanding leaf morphogenesis must also include deciphering the rules governing the development of venation systems. The general mechanism has been recently described and auxin transport and distribution have been recognized as the central factors responsible for venation patterning. This issue has been extensively reviewed in Scarpella, Barkoulas, and Tsiantis [45] and, therefore, we will not discuss it here. From the physiological point of view, veins are important for water and nutrient distribution across the leaf. From the structural and biophysical perspective, they support the leaf, which allows for development of certain shapes and sizes of this organ. From the engineering point of view, the most appropriate venation pattern ensures a high transport capacity relative to construction cost therefore, a certain degree of similarity is shared even between distant plant species. A quantitative description of the venation patterns in *Arabidopsis thaliana* leaves has been recently performed with help of the LIMANI open-source *in silico* tool [46]. This work showed that the pattern of vein branching in *Arabidopsis* is independent from the developmental stage and cellular content, suggesting the existence of a self-organizing regulatory mechanism. The existence of such a self-regulating system in which pattern accommodates to form has been demonstrated experimentally by local changes in growth distribution triggered by expression of the *KRP1* gene in the CUC2 domain [47]. When obtained this way in deeply dissected leaves, (see next part of this review) the veins accommodated drastic changes in leaf shape but no major differences in general rules governing venation pattern have been observed. The LIMANI tool showed also that there are differences in venation density across subsequent leaves within the rosette. Surprisingly however, the opposite trend has been observed between two different ecotypes (Col0 and Ler), thus reflecting impact of the genotype. In *Arabidopsis*, different shapes of subsequently developed leaves are related to the vegetative phase change and this process is under strict molecular regulation [48,49]. We believe that there may be some discrete differences in regulatory mechanisms between these two ecotypes.

**Figure 3:**
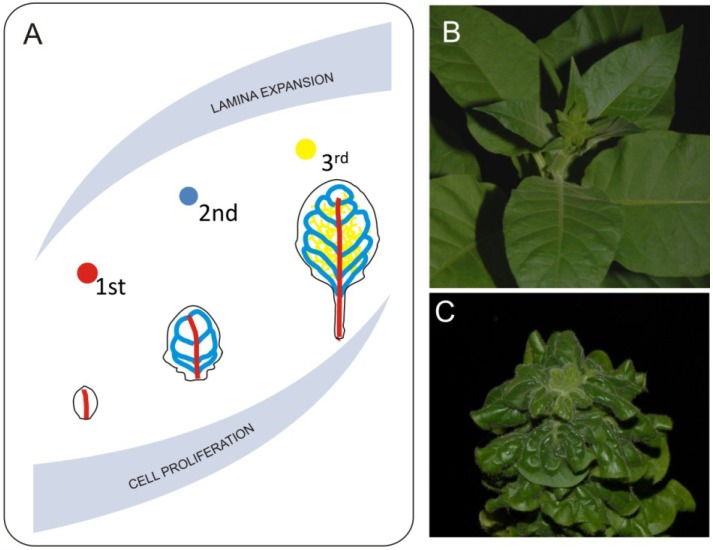
Mechanical effects of venation systems on leaf growth. Midvein and second degree veins differentiate during early stages of leaf development. This event overlaps with proliferative phase of leaf growth (**A**). Such situation has huge consequences on leaf morphology since 1st and 2nd degree veins reach they pattern at early stages of leaf development and will not accommodate it during further leaf expansion. Further degree veins differentiate during expansive phase of leaf development and they follow the lamina growth. Direct consequence of disturbed coordination between leaf blade expansion and growth of venation system is extreme 3-D deformation such as observed in the *bri1* brassinosteroid mutant plants. Pictures show comparison between morphology of properly developing (**B**) and brassinosteroid insensitive (**C**) tobacco plants.

Another observation made with the LIMANI tool is that during the exponential phase of leaf growth, the vascular pattern extends faster than the size of leaf blade, whereas later on the differentiation of new veins slows down. Since differentiation of major veins starts during the transition from primary to secondary leaf morphogenesis, both cell cycle and cell growth will have an impact on certain aspects of venation (Figure 3). Work by Dhondt *et al*. [46] shows that change in the timing of cell proliferation affects venation. They also show that discrete differences in growth dynamics between veins and parenchyma cells occurs—for example, parenchyma cells inside the differentiated areole still grow, influencing the final areole size. Lack of proper growth coordination between veins and other components of the lamina heavily influences leaf development. One example of such a disturbance is the brassinosteroid receptor mutant *bri1*. Leaves of *bri1* mutants are small with a dark green lamina caused by decreased cell growth [24]. Another very striking feature of these leaves is a curly phenotype most probably caused by disturbed coordination of growth between veins and lamina (Figure 3). Veins of the *bri1* mutant are thicker in diameter but shorter than the corresponding veins in wild type plants. Disturbance in brassinosteroid synthesis or perception affects both cell growth and divisions and most probably leads to disturbances in the mechanism regulating proper growth distribution between lamina and veins [24,50,51]. This disproportion generates tensions that result in organ deformations. Veins of the *bri1* mutant are less resistant to bending and it is easier to break them. If we mechanically remove the midvein by excision from a curly leaf we can expand the resulting pieces of the lamina. This shows how lack of growth coordination between major veins and the lamina leads to three-dimensional deformation. Possible impacts of mechanical forces in this process should also be taken into account. *In silico* modeling suggests that generation of certain aspects of vein patterning may be related to a mismatch in elastic properties between different cellular layers of the leaf [52], therefore one can imagine that a disturbance of precise cellular coordination may lead to major changes in the venation system. Another recently completed modeling shows that higher degrees leaf venation may rely on the distribution of mechanical tensions within the organ [53]. To our knowledge, thus far, high resolution studies describing the mechanical impact of main veins on leaf development have not been published, however theoretical aspects of such 3-D leaf deformation was discussed [2].

Comparisons of leaves from different species shows that certain mechanical features of veins and venation patterns correspond to adaptations of plants to different environmental conditions. For example, plants from extremely dry habitats have smaller leaves with higher major vein density, which helps to decrease leaf hydraulic vulnerability [54]. Recently completed comparative studies across 485 plant species, differing in leaf size, shape, venation patterns, and habitats, brought a very clear picture of the correlation between leaf anatomy and adaptation of plants to different environments [55]. Sack *et al*. [55] also found that higher degree veins do not contribute to leaf size, which is in agreement with previous modeling works [53]. Based on these studies, we may expect that modification of major veins’ growth or patterning or change in their biomechanical properties could be a very promising direction for future engineering of plant architecture.

## 8. Gradients of Growth and Leaf Shape Formation

Higher plants developed a large variety of leaf forms differing in size and shape. There has been much speculation as to how leaf shape contributes to plant life strategies; however recent review by Nicotra *et al*. [56] suggested that it can be an important trait influencing water relations. In the light of recently published work by Sack *et al*. [55] this statement is fully justified (see chapter above). Nicotra *et al*. [56] also mention that leaf shape variation is not an independently functional trade-off, instead it can work together with different traits (e.g., leaf size, number, stomatal conductance) as an additional factor allowing for better adjustment to the environment. Further exploration of leaf shape regulatory mechanisms and their connections with other traits by precise modification of leaf shape has future potential in generation of the energy efficient or environmentally adapted plants.

The final leaf shape is a result of interplay between growth rate distribution and growth orientation. Recent work showed that to a large extent across distant plant species these two parameters are modulated by the NAM/CUC transcription factors [57]. The recruitment of the same molecular factors, however, results in various leaf forms. Therefore, deciphering rules governing leaf shape acquisition needs an interdisciplinary approach.

The model describing interplay between NAM/CUC transcription factors and auxin has been recently completed [58]. According to this work, generation of auxin maxima on the leaf margin and subsequent directional auxin transport is responsible for lobe outgrowth, whereas NAM/CUC factors are involved in promotion of PIN1-dependent auxin transport, thus defining the area of local tissue outgrowth. Auxin was also shown to suppress *CUC2* gene expression and restrict it to the sinus area [58]. This shows that the main factor influencing lateral leaf outgrowths (lobes, serrations, *etc.*) is auxin; on the other hand, this suggests that CUC2 may specifically restrict growth. The cellular basis of this growth restriction has not been fully verified; however, the work of Kawamura *et al*. [59] suggests that increased cell proliferation is present in the sinus area. This result shows that differential growth within the leaf lamina strongly depends on the timing of cell proliferation and increased or prolonged proliferation may in fact lead to net growth reduction. This phenomenon has been also observed when *CYCD3.1* G1-S progression factor was overexpressed in *Arabidopsis* leading to generation of a smoother and smaller leaf blade compared to the corresponding leaf in undisturbed plant [29].

The first visible signs of leaf dissection overlap with transition from cell proliferation to differentiation [39], therefore, we may say that regulation of meristematic activities also affects final leaf shape. Important factors regulating the cell proliferation status in leaves are TCP genes, and disturbance of their action leads to serious defects in leaf expansion and shape acquisition [60,61]. It is worth mentioning that some members of the TCP group negatively regulate cell proliferation whereas others are positive regulators. Another group of genes of which activity has been linked to meristematic capacity are KNOX transcription factors. Typically, their activity is excluded from leaves, however in some species the appearance of *KNOX* gene expression on the leaf margin leads to compound leaf development. One such example is *Cardamine hirsuta*—a close relative of *Arabidopsis*. Leaves of *Cardamine* are compound due to the promotion of the meristematic state of cells by occurrence of local KNOX gene activity within the margin area [62]. Barkolaus *et al*. [63] showed that the occurrence of these local areas of KNOX activity is regulated by auxin distribution. *KNOX* gene expression is excluded from non-compound blades of *Arabidopsis* and variations of *KNOX* expression in leaves of different relatives of *Arabidopsis* influence the degree of leaf dissection, thus reflecting evolutionary change [64].

Recent work on tomato plants has shown that cytokinins also regulate leaf shape, mainly by the regulation of cell proliferation, timing, and maintenance of morphogenetic capacity within the margin area of the developing leaf [65]. Some effects of cytokinin activity are probably linked to modulation of auxin transport whereas others involve the direct influence of cytokinin on cell cycle progression.

**Figure 4:**
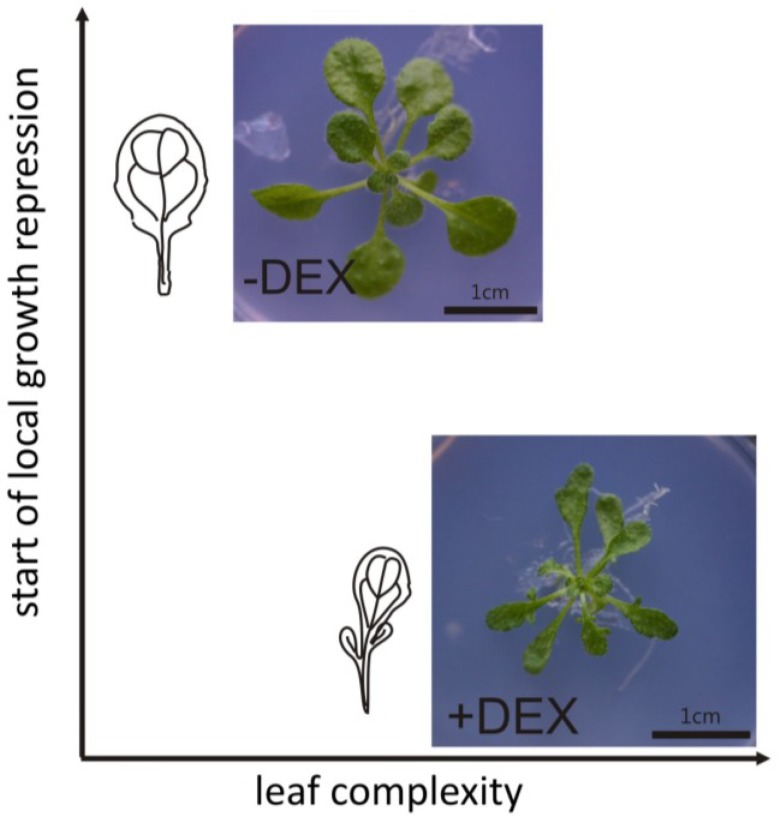
The effect of local growth repression on leaf complexityGeneration of local decrease in growth during early stages of leaf development results in increased leaf dissection. The pattern of these local gradients of growth distribution is established during proliferative phase of leaf growth and later manipulation does not result in any major change in leaf complexity. Figure shows effects of local growth arrest induced by chemical induction of the *KRP1* gene within the *CUC2* expressional domain in *Arabidopsis* thaliana plants (−DEX before and +DEX after induction). Figure based on Malinowski *et al*. [47].

The molecular framework regulating leaf shape determination differs between species. However, with the exemption of plants whose leaf dissection is generated via programmed cell death [66], most leaf forms need an early generation of gradient of growth distribution along the margin. Work with *Cardamine* shows that local meristematic activity and further maintenance of cell proliferation leads to the development of compound leaves, whereas leaf serration (e.g., in *Arabidopsis*) depends only on the maintenance of cell proliferation during lobe outgrowth. The manipulation of local gradients of growth promotion and repression within the leaf margin shows however that leaf dissection may also be generated by local growth repression [47]. Artificial manipulation of lamina growth by targeting the *KRP1* cell cycle inhibitor expression to the *CUC2* expressional domain led to the development of deeply serrated leaves (Figure 4) [47]. Since no change in native *CUC2* gene expression pattern was observed, this manipulation most probably did not affect the formation of auxin convergence points, therefore the observed deep serrations were generated exclusively by imposing local growth restrictions to an otherwise growing system. An increase in *KRP1* gene expression around the margin area led to the suppression of growth gradients, resulting in the arrest of lobe outgrowths and subsequent 3-D leaf blade deformation. This work shows that auxin-related local outgrowth and the timing of local growth repression both influence leaf complexity and a balance between these two factors may reflect the existence of different leaf forms.

Recently, a general model describing lamina growth, which takes into account factors influencing lateral outgrowth as well as determination of the proximo-distal polarity, has been published [67]. Combinations of time-lapse imaging, clonal analysis, and computational expertise resulted in the generation of a robust model sufficient to describe the development of distinct leaf shapes. Similar to the experiment with local expression of the *KRP1* gene [47], this model clearly shows that manipulation of growth distribution during early stages may influence final leaf shape, whereas modifications during late stages do not heavily influence leaf patterning and growth rates except in regions in close proximity to the manipulation. A model created by Kuchen *et al*. [67] describes general mechanisms of growth coordination during leaf shape acquisition. The authors tested several combinations of factors and derived a theoretical model of interactions which can be used to describe generation of overall leaf shapes such as oblong, cordate, ovate, elliptic, *etc*. This model reflects growth rates, growth distribution and growth directions revealed on time-lapse observations of leaf growth. This work resulted in the formation of an “Organizer-based” model, which takes into account two major regulatory networks—first specifying the growth polarity and second regulating the growth rate. According to the model, the synthesis of factor regulating growth polarity is promoted at the proximal base of leaf primordia by the so-called proximal organizer. Furthermore, a gradient of the polarity-regulating factor is maintained by its constant degradation within the remaining part of primordium. In order to reflect growth directions observed *in planta*, the authors determined that distribution of the factor regulating growth polarity deforms together with the organ during subsequent growth. This polarity network is strictly connected with networks regulating growth rate. The authors distinguished two types of growth rates, parallel to the primordium midline, and perpendicular to the midline. In later steps of primordium development, these main growth rates are influenced by the production of uniformly distributed proximodistal growth inhibitors, resulting in a higher perpendicular growth rate. Additionally, perpendicular growth rate is positively regulated by another factor whose distribution is gradual in proximodistal direction and is excluded from the base of primordium. In order to reflect the growth directions in a presumptive midvein area, the authors placed another factor, which negatively influences the perpendicular growth rate in the midline of the leaf primordium. Spatial and temporal modifications of particular elements of this theoretical set up allows generation of various, biologically relevant leaf shapes. At present, we do not know exactly which protein, gene, morphogen, *etc*. represents the particular factor designed by the authors. It is only a theoretical model, however it may work as a framework for further experimental investigations; and predicted interactions may help to illustrate the interactions between different regulatory networks influencing final leaf form. A model created by Kuchen *et al*. [67] shows that the general rules governing leaf form specification may be shared across distinct plant species, however in order to understand how different leaf forms are generated, further detailed studies on molecular mechanisms determining cellular parameters in particular species are necessary.

## 9. Concluding Remarks

The results presented in this review reveal the technical progress that has occurred over the last decade, allowing us to verify long-standing hypotheses. The development of modern imaging methods and computational tools has helped us to study dynamic cellular changes during leaf development. This work resulted in the discovery of mechanisms involved in the precise coordination between mechanical inputs and growth responses during plant organogenesis [14,15]. Today, we can be sure that the biophysical level is a very important component of global regulation of plant organogenesis. In case of leaves, there is still much work that must be done in order to link the mechanisms regulating cell proliferation/differentiation balance with biophysical inputs. Precise measurements and cell tracking techniques showed that there are multiple levels of regulation influencing final leaf form [29,32,35,36,37]. This information is still incomplete, and further work needs to be done in order to characterize all complex cellular relations. Finally, use of chemically inducible expression systems allowed us to show directly that local changes in growth are responsible for the generation of complex leaf shapes [47]. Integration of these recent findings with patterns of auxin transport and genetic networks may be additionally supported by a recently generated theoretical model describing overall leaf shape generation [67]. The holistic perspective of these studies already resulted in the discovery of general rules governing leaf size and shape acquisition, but future understanding of how exactly particular leaf forms are generated will need further characterization of genetic factors and their involvement in coordination of all regulatory levels. The use of precise systems for modification of gene expression will ultimately help to test the exact involvement of newly characterized factors in a defined biological context. As one can clearly see, further progress in understanding of these complex biological subjects requires an integrative approach. There are already multiple examples where advanced equipment designed for medical or purely physical applications such as X-ray tomography has been used for plant biology studies [68]. We believe that this tendency will continue and further integration of scientific methods will result in an understanding of leaf development at the system level.
